# Statistical Analysis of Gastric Cancer Cells Response to Broadband Terahertz Radiation with and without Contrast Nanoparticles

**DOI:** 10.3390/cancers16132454

**Published:** 2024-07-04

**Authors:** Oliver Daniel Schreiner, Diana Socotar, Romeo Cristian Ciobanu, Thomas Gabriel Schreiner, Bogdan Ionel Tamba

**Affiliations:** 1 Department of Electrical Measurements and Materials, Gheorghe Asachi Technical University, 700050 Iasi, Romania; oliver090598@yahoo.com (O.D.S.); diana.socotar@gmail.com (D.S.); 2 CEMEX-Center for Experimental Medicine, “Grigore T. Popa” University of Medicine and Pharmacy, 700259 Iasi, Romaniabogdan.tamba@umfiasi.ro (B.I.T.)

**Keywords:** broadband terahertz spectroscopy, cancer cells detection, contrast nanoparticles, statistical analysis, Wilcoxon signed-rank test

## Abstract

**Simple Summary:**

The paper describes the statistical analysis of the response of gastric cancer cells and normal cells to broadband terahertz radiation up to 4 THz, both with and without the use of nanostructured contrast agents. A statistical analysis was conducted towards multiple pairwise comparisons in order to identify the narrow THz frequency ranges where the contrast is enhanced and allows a clear identification of tumor cells in the presence of contrast agents.

**Abstract:**

The paper describes the statistical analysis of the response of gastric cancer cells and normal cells to broadband terahertz radiation up to 4 THz, both with and without the use of nanostructured contrast agents. The THz spectroscopy analysis was comparatively performed under the ATR procedure and transmission measurement procedure. The statistical analysis was conducted towards multiple pairwise comparisons, including a support medium (without cells) versus a support medium with nanoparticles, normal cells versus normal cells with nanoparticles, and, respectively, tumor cells versus tumor cells with nanoparticles. When generally comparing the ATR procedure and transmission measurement procedure for a broader frequency domain, the differentiation between normal and tumor cells in the presence of contrast agents is superior when using the ATR procedure. THz contrast enhancement by using contrast agents derived from MRI-related contrast agents leads to only limited benefits and only for narrow THz frequency ranges, a disadvantage for THz medical imaging.

## 1. Introduction

Cancer is among the most prevalent diseases in contemporary society, exerting a substantial influence on the quality of life, straining health budgets, and profoundly affecting families emotionally. Due to its insidious onset, rapid and nonspecific dissemination throughout the body with the formation of metastases, variable reactivity to oncolytic therapy, and ability to evade immune responses, neoplastic formations are often detected at an advanced stage of carcinogenesis. Gastric cancer, in particular, continues to pose a significant global challenge, exhibiting a high mortality rate of 95% when diagnosed at an advanced stage (IV TNM). In recent years, stomach cancer has been estimated to be the third leading cause of cancer-related mortality globally for both sexes, accounting for 723,000 deaths or 8.8% of the total. Gastric tumors are frequently diagnosed at advanced stages, with at least 17% of cases progressing to metastasis. The capability to meticulously visualize the mucosa of the gastrointestinal tract is essential for the early detection of dysplasia and cancer. Although techniques such as chromoendoscopy, high-resolution and magnification endoscopy, narrow-band imaging, and autofluorescence imaging may enhance the visualization and detection of mucosal lesions, a biopsy of the targeted lesion remains essential for a definitive histological diagnosis of cellular and architectural atypia. Recent technological advancements in miniaturization have facilitated the integration of a confocal scanning microscope into a conventional flexible endoscope, a method now referred to as confocal endomicroscopy (CEM). This newly developed technology allows endoscopists to acquire real-time in vivo histological images of the gastrointestinal mucosa during an endoscopy, generating significant interest in its application within clinical gastroenterology. However, to obtain high-contrast images during fluorescence-based confocal endomicroscopy (CEM), intravenous and topical contrast agents such as fluorescein, acriflavine, tetracycline, and cresyl violet are essential. Among these contrast agents, intravenous fluorescein sodium (10%) and topically applied acriflavine (0.2%) are the most commonly used in humans. Consequently, although rare, adverse reactions to these contrast agents, including serious effects such as anaphylaxis or cardiac and respiratory complications, may occur. Overall, a widely accessible and perfect methodology for the early detection of gastric neoplasia remains an unmet goal [1,2,3,4]. Recently, terahertz radiation has garnered attention due to its combined detection capabilities, along with its non-invasive and non-ionizing properties. The principle of the terahertz (THz) test employs surface plasmon polaritons instead of photons as the exposure source. This approach can pattern nanoscale feature sizes due to its near-field enhancement effects. An innovative test methodology grounded in basic and clinical research, emphasizing terahertz molecular imaging as an advanced early-stage diagnostic tool, could serve as an interdisciplinary approach in the field [5,6,7,8,9,10,11,12,13,14,15,16,17,18,19,20,21,22,23]. Significant research efforts in this area commenced around 2010, and it remains an evolving imaging technology for the localization of cancer tumors [24,25]. Nanotechnology presents opportunities for innovation in medical imaging by enabling the creation of new contrast agents that enhance image resolution in areas where normal and pathological tissues coexist. While this realm of investigation remains nascent, the initial findings outlined in the literature exhibit promise, paving the way for novel prospects in the clinical utilization of MRI and potentially THz medical imaging. A novel concept involving contrast nano-carriers tailored for the THz domain could be envisioned with the following attributes: biocompatibility, an affinity for cancerous tissue, stability, efficiency, and facile elimination within a 24 h timeframe. These particles should be readily injectable or dispersible, with a prerequisite of non-coagulation and non-reactivity with gastric acid and other pertinent biological agents. Studies in this field have focused on highly dispersed nanostructured materials, including metallic (e.g., gold nanorods), magnetic (e.g., derived from Fe_2_O_3_ or Fe_3_O_4_), and nano-diamonds or dedicated metamaterials. However, there is a lack of precise definitions regarding the potential characteristics required to enhance image contrast or resolution [26,27,28,29,30,31,32,33,34,35]. A strategy involving coupling with monoclonal antibodies could be combined with passive accumulation via the enhanced permeability and retention effect to attain the active retention of nano-assemblies. Therefore, the advancement and application of customized nanoparticles to augment specificity and amplify the resolution of THz molecular imaging up to the micro-scale could signify a significant breakthrough. In recent years, the progression of such contrast agents has stemmed from those conventionally employed for MRI, such as assemblies incorporating gadolinium oxide, as described in the literature [36,37,38,39,40], yielding notably superior outcomes. Some of these agents have been effectively tested for THz imaging. Hence, gadolinium diethylene glycol penta-acetic acid (Gd-DTPA) emerges as a promising candidate for THz imaging despite not being tested in this context. It has garnered significant interest, owing to its potential for targeted cellular localization through antigen or antibody attachment. Furthermore, it has demonstrated low toxicity and is readily eliminated via renal excretion due to its low molecular weight. The prominent techniques in THz imaging encompass electro-optic (EO) imaging, single-shot imaging, close-field imaging, dark-field imaging, bistatic THz wave imaging, THz wave computed tomography (CT), and THz wave tomographic imaging employing Fresnel lenses. Presently, THz pulse imaging, THz time-domain spectroscopy (TDS-THz), and continuous wave terahertz (CW-THz) are recognized as the most promising methodologies for biomedical applications, particularly when combined with innovative contrast agents capable of augmenting image contrast and selectivity. In the context of the differential mode, it is theoretically anticipated that the THz signal originating from cancer cells with contrast agents would be up to 30 times greater than that of cancer cells lacking nanoparticles. However, experimental evidence has not validated this projection, as observed increases have not surpassed a factor of 3. Nevertheless, the ultimate objective for advancing an innovative THz endoscopic approach necessitates achieving excellent contrast and attaining a resolution in the range of a few microns. This is crucial for enhancing THz endoscopy’s feasibility, specificity, and sensitivity. Some highly capable THz imaging devices, albeit not exclusively designed for biomedical applications, have already been introduced into the market or are in the pipeline for release, as documented in [41]. These devices hold the potential to enhance THz imaging for cancer diagnostic purposes. Despite accurately predefining the parameters of the tested samples and efficiently utilizing the software package along with its features for enhancing contrast or resolution, as outlined in [42,43,44], there exist distinct limitations concerning the evaluation of the THz methodology, both in transmission and reflection modes, particularly when employing a narrow frequency range of 0.5–1.8 THz. The contemporary scholarly literature delineates spectral inquiries conducted on a diverse array of noteworthy biological specimens. These investigations aim to elucidate the interaction between THz radiation and biological systems at the molecular level, focusing on the particular resonance processes governing the electronic, vibrational, and rotational behaviors of intricate biological molecules. By virtue of variances in water content and structural configurations within tissues, normal and neoplastic structures manifest distinct THz absorption characteristics. Cancerous tissue, characterized by heightened vascularization or edema, harbors greater interstitial water content, as corroborated by findings from PET, MRI, and similar modalities. A significant challenge in THz imaging pertains to the interaction of wave energy with substances, particularly within the biological milieu under analysis. Significant investigations have, to some extent, substantiated the viability of the concept for detecting cancer cells (from the breast, colon, stomach, and larynges), epithelial cells, or corneal tissues, both in vitro and, to a lesser extent, in vivo [45,46,47,48,49,50,51,52,53,54,55,56,57,58,59,60,61,62,63], utilizing THz spectroscopy.

A recent development in THz analysis for the biomedical field involves reassessing parameters associated with broadband THz spectroscopy, as evidenced in [64,65,66,67,68,69,70,71,72,73,74,75]. Additionally, there is a trend towards broadening the frequency range of THz analysis for studying cancer phenomena, extending from 1.8 THz, as outlined in [76], to 4 THz and beyond, as explored in [77,78,79]. To achieve this objective, Terahertz-attenuated total reflection (THz-ATR) spectroscopy is frequently employed, as exemplified in [80], due to its perceived higher sensitivity compared to the aforementioned methods. Conversely, contemporary interpretations of THz spectroscopy parameters involve the utilization of mathematical techniques, machine learning algorithms, or artificial intelligence for automated recognition, as discussed in [81,82,83,84,85].

Aligned with the current trends in THz signal analysis, this study aimed to examine the response of cancer cells to broadband terahertz radiation up to 4 THz, an aspect not found in the literature. This frequency range is considered optimal for the development of biomedical applications. The investigation was conducted both with and without the use of contrast agents. The THz spectroscopy analysis was comparatively performed under the ATR procedure and transmission measurement procedure. The novelty of the paper consists in proposing a new statistical method in order to identify the most appropriate narrow bands in the THz domain by analyzing the behavior of cells in the broadband THz domain and accounting for their optimal response. The presented research could help physicians in developing and testing new contrast agents, mainly due to the fact that not all the current contrast agents, e.g., in use for MRI, can be automatically used for THz imaging in medical practice.

## 2. Materials and Methods

### 2.1. Characterization Equipment

**Figure 1:**
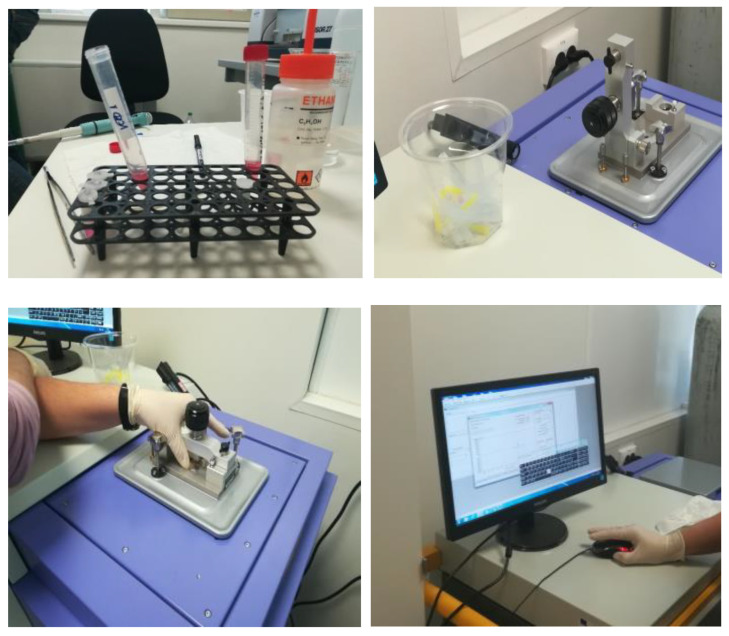
Operational stages of the spectroscopy in the THz field.

THz spectroscopy and imaging are continuously progressing as robust technique for different applications in the field of medicine, mainly related to oncology. THz radiation is non-ionizing and, associated with safe energy levels, has the potential to achieve high-resolution data related to cells or tissues, effectively combining both macroscopic and microscopic information. In our research, THz spectroscopy was conducted utilizing the TeraPulse Lx equipment (TeraView, based in Cambridge, UK). The procedural steps involved in the THz spectroscopy methodology up to 4 THz encompassed sample preparation for inclusion in the measurement cell, securing the measurement cell, device calibration, and subsequent data collection and processing, as illustrated in Figure 1. 

### 2.2. Contrast Agents Description 

**Figure 2:**
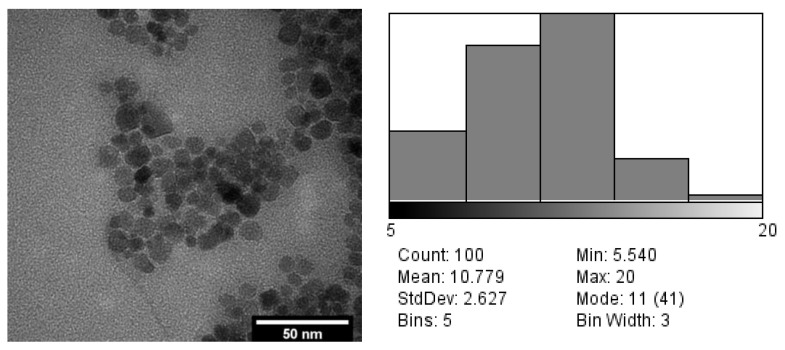
TEM micrograph of Gd_2_O_3_ functionalized with carboxymethylcellulose and particle size distribution.

The contrast agent utilized was a composite of gadolinium oxide, which was synthesized by functionalizing commercial gadolinium oxide with carboxymethylcellulose. This process was adapted from methodologies employed for superparamagnetic iron oxide, as documented in [38,40,43]. Transmission electron microscopy (TEM) analysis, conducted using the JEOL 2100 Plus equipment from JEOL Ltd., located in Akishima, Tokyo, Japan, as depicted in Figure 2, revealed that the functionalization process facilitated the complete disaggregation of inorganic particles in the presence of the polymer under consideration, resulting in an average size of 10.78 nm [38,40]. The consideration of this aspect is pivotal when assessing the interaction with the cellular medium. The primary benefits include the straightforward and rapid preparation of the compounds, reproducibility of the chemical composition, and precise control over the particle size distribution through the modulation of the parameters within the co-precipitation process.

Advancements in creating safe and efficient THz contrast agents could significantly enhance image quality by amplifying the contrast between normal and diseased tissues. However, simply converting MRI-related contrast agents towards THz analysis may not fully respond to this purpose, even if it is the simplest way of achieving medically accredited contrast agents. 

### 2.3. Test Cells Description 

Cell line 1: AGS cell line (human gastric adenocarcinoma cell line, CLS 300408). 

Cell line 2: Hs 738.St/Int cell line (human gastric fibroblast cell line, CRL-7869). 

Each cell line was cultured in Dulbecco’s modified Eagle’s medium (DMEM, Biochrom AG, Berlin, Germany), supplemented with 10% fetal bovine serum (FBS, Biochrom AG, Germany), 100 μg/mL streptomycin (Biochrom AG, Germany), and 100 IU/mL penicillin (Biochrom AG, Germany). The cell cultures were then seeded in 12-well plates at a density of 20,000 cells per well and maintained in a CO_2_ incubator at 37 °C. 

**Figure 3:**
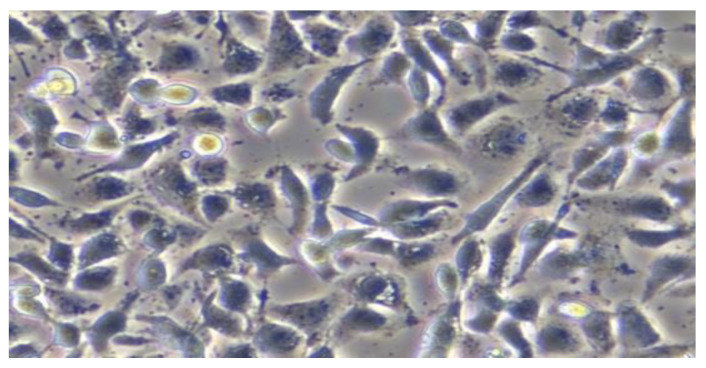
Morphology of cells treated with nanoparticles (diluted 1:5 in DMEM).

Following the removal of the medium and washing of the cells, a fresh, complete medium supplemented with functionalized Gd_2_O_3_-carboxymethylcellulose nanoparticles was added at a dilution of 1:5, which represented the maximum feasible dilution for an enhanced effect. The selection of dilutions was based on a prior assessment of cell viability using the MTT assay. The treatment duration lasted for 24 h, and upon completion of the treatment period, the plates were examined using phase-contrast microscopy (Nikon TS2 inverted microscope, 200× magnification – Nikon Corp., Tokyo, Japan). The investigation focused on assessing changes in cell morphology and density, as well as evaluating the biocompatibility of the nanoparticles, as referenced in [37,40,42]. No notable cell morphology or density alterations were observed, as illustrated in Figure 3. 

Before being submitted to the analysis under THz radiation, cell viability dynamics was determined by MTT assay after 24 h incubation of cells in the presence of different dilutions of nanoparticles. The results obtained indicate the existence of a much higher degree of tolerance in the case of normal cells compared to tumor cells, due to their functional capacity to respond adequately to xenobiotic stressors, but in both cases, the degree of viability was extremely high, of over 80%, validating the use of the proposed contrast nanoparticles. 

## 3. Results and Discussion

### 3.1. ATR Analysis

**Figure 4:**
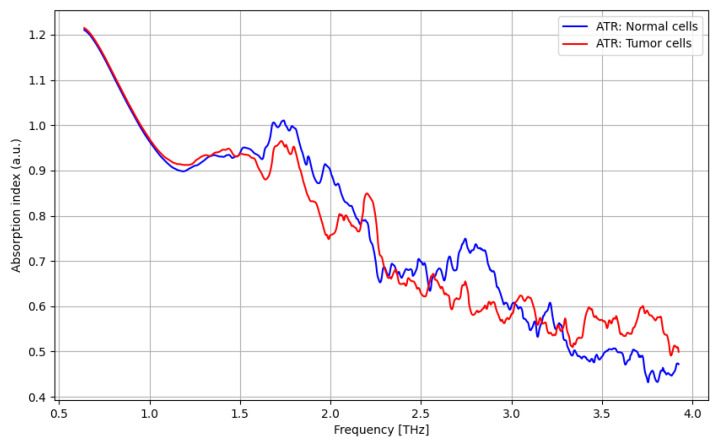
ATR/absorption characteristics for tumor cells and normal cells.

**Figure 5:**
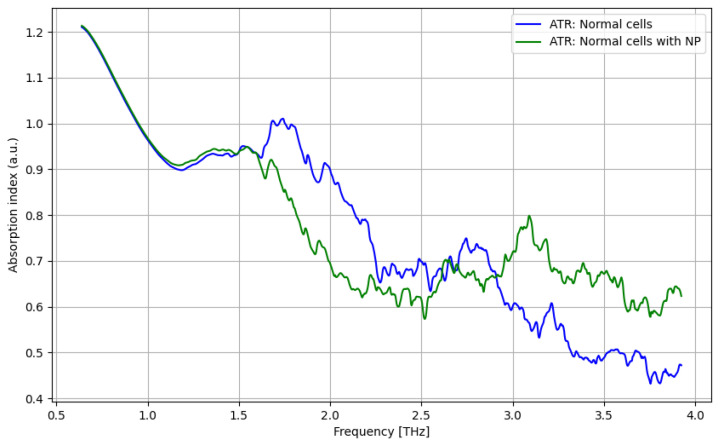
ATR/absorption characteristics for normal cells and normal cells with nanoparticles.

**Figure 6:**
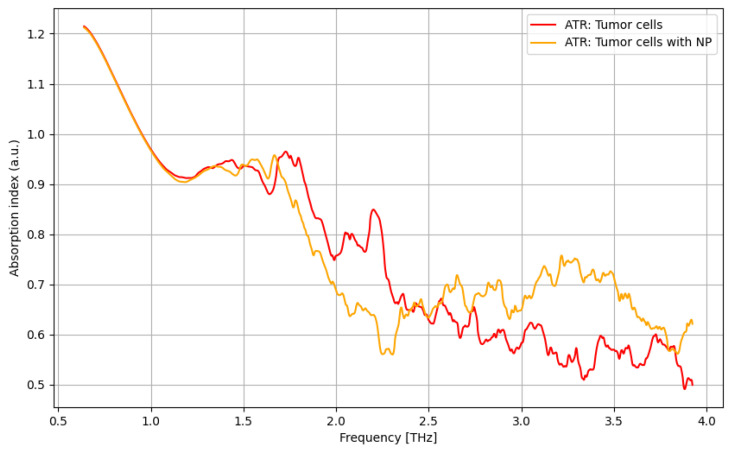
ATR/absorption characteristics for tumor cells and tumor cells with nanoparticles.

**Figure 7:**
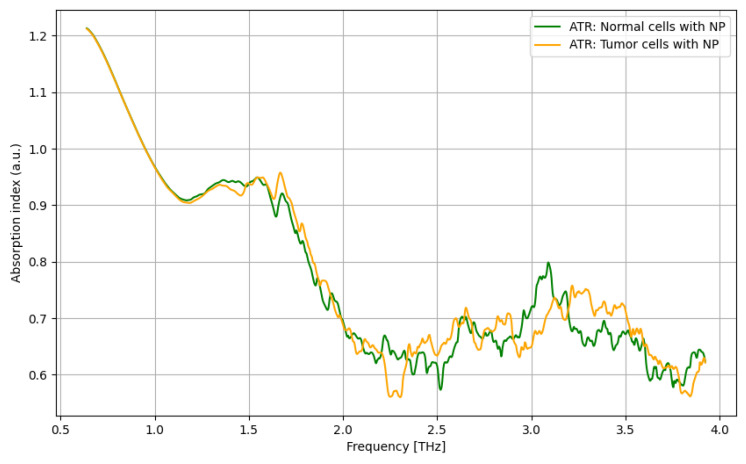
ATR/absorption characteristics for normal cells with nanoparticles and tumor cells with nanoparticles.

The THz spectroscopy analysis under the ATR procedure is presented vs. the applied frequency (0.06–4 THz) in Figure 4, Figure 5, Figure 6 and Figure 7. As presented in Figure 4, a significant difference between tumor and normal cells can be noticed for the frequency domain of 2.5–3 THz, even if the difference is low. As presented in Figure 5, a significant improvement in identifying normal cells using contrast nanoparticles can be achieved for the frequency domain of 3–4 THz, with a relevant difference. 

As presented in Figure 6, a significant improvement in identifying cancer cells using contrast nanoparticles can be achieved for the frequency domain of 2.75–3.75 THz, with a relevant difference. 

Finally, as presented in Figure 7, the highest discrimination between tumor cells and normal cells by using nanoparticles may be noticed mainly in the frequency domain of 3.25–3.5 THz, even if the differentiation can be extended from 2.25 to 3.75 THz. 

**Figure 8:**
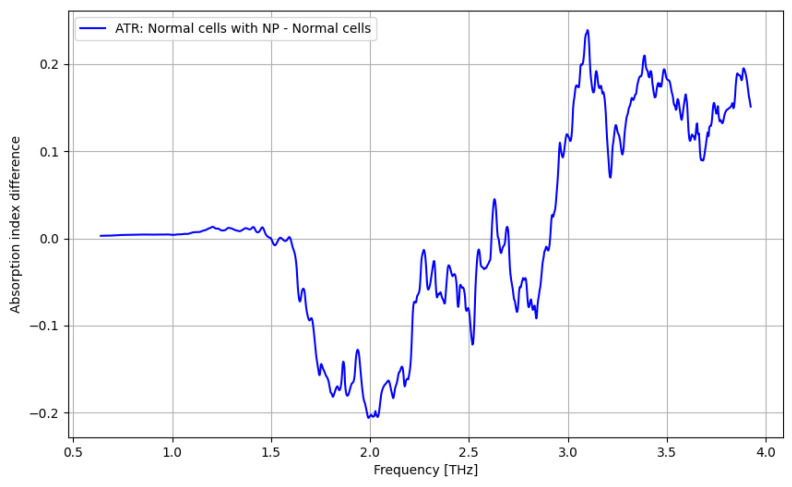
ATR/absorption enhancement for normal cells by use of nanoparticles.

In Figure 8, the contrast enhancement by use of nanoparticles is presented in terms of the identification of normal cells. The most relevant frequency domain is over 3 THz.

**Figure 9:**
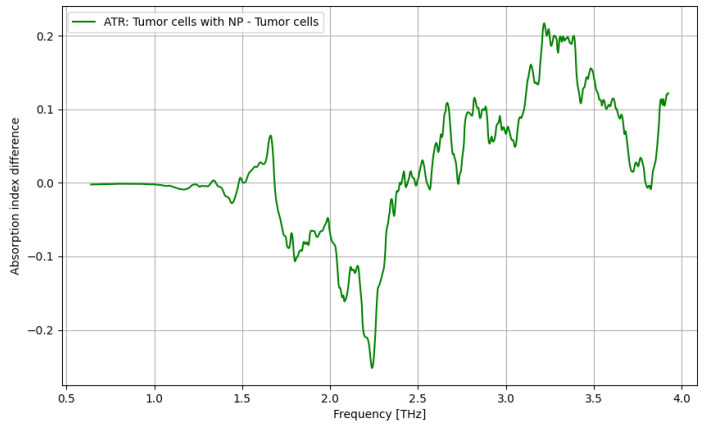
ATR/absorption enhancement for tumor cells by use of nanoparticles.

In Figure 9, the contrast enhancement by use of nanoparticles is presented in terms of the identification of tumor cells. The most relevant frequency domain is 2.75–3.75 THz.

### 3.2. Transmission Analysis

**Figure 10:**
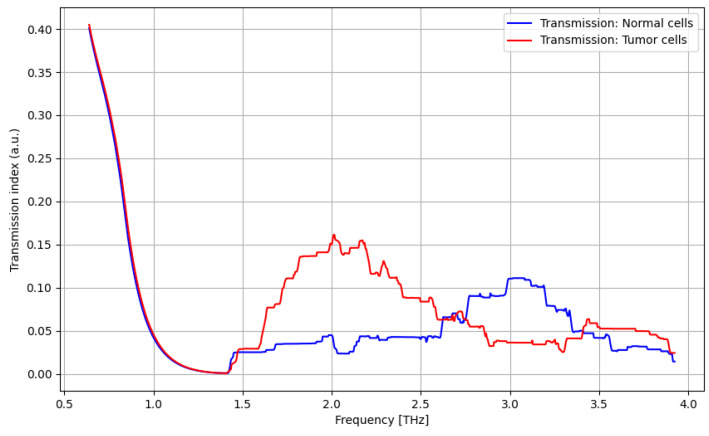
Transmission characteristics for tumor cells and normal cells.

**Figure 11:**
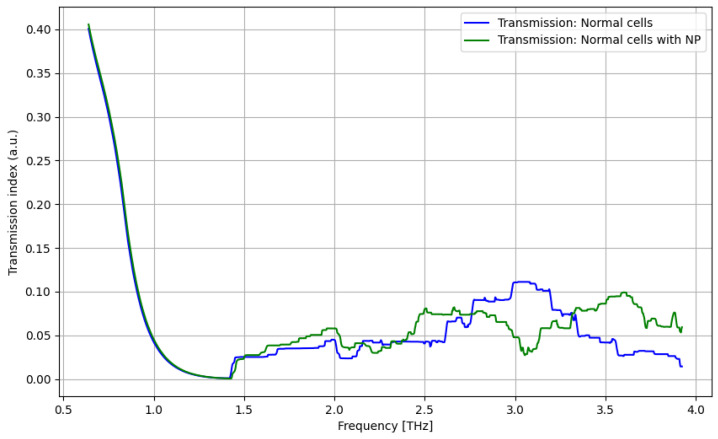
Transmission characteristics for normal cells and normal cells with nanoparticles.

**Figure 12:**
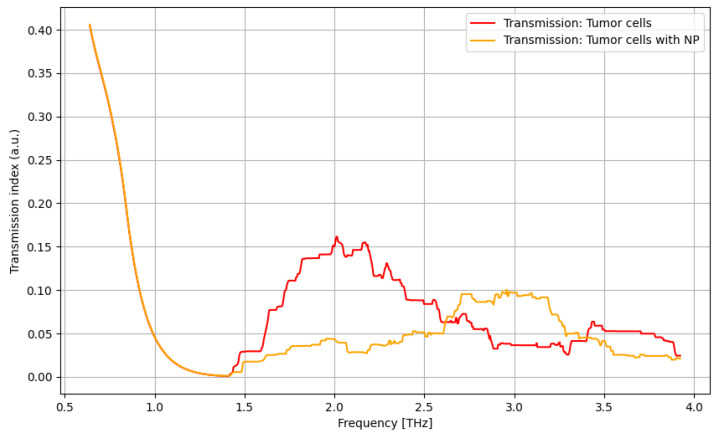
Transmission characteristics for tumor cells and tumor cells with nanoparticles.

**Figure 13:**
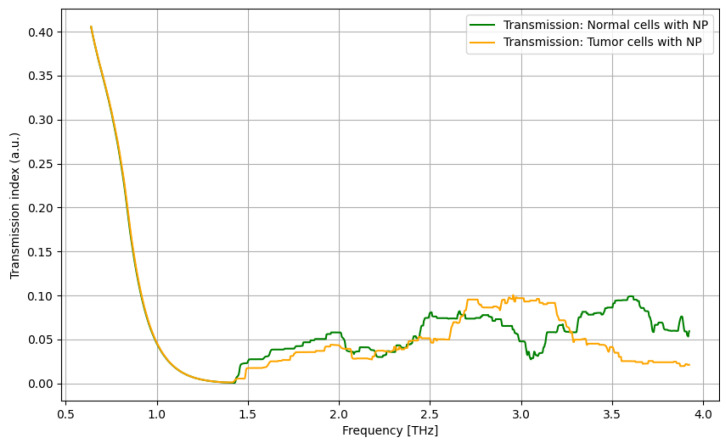
Transmission characteristics for normal cells with nanoparticles and tumor cells with nanoparticles.

The analysis of the transmission of biological samples in the THz domain (0.06–4 THz) is presented in Figure 10, Figure 11, Figure 12 and Figure 13. 

As presented in Figure 10, a significant difference between tumor cells and normal cells can be noticed for the frequency domain of 1.5–2.5 THz, with a relevant difference. 

As presented in Figure 11, a significant improvement in identifying normal cells by using contrast nanoparticles can be achieved only at a frequency domain of over 3.25 THz. 

As presented in Figure 12, contrast nanoparticles can significantly improve the identification of cancer cells in the frequency domain of 1.5–2.5 THz, with a relevant difference and with lower relevance at over 3.4 THz. 

**Figure 14:**
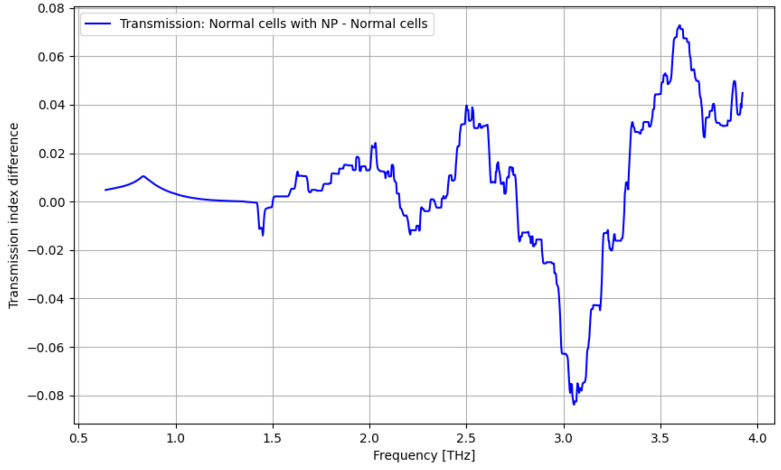
Transmission signal enhancement for normal cells by use of nanoparticles.

Finally, as presented in Figure 13, the highest discrimination between tumor cells and normal cells by using nanoparticles may be noticed mainly in the frequency domain of 2.6–3.25 THz. In Figure 14, the contrast enhancement by using nanoparticles is presented in terms of identifying normal cells. The most relevant frequency domain is over 3.5 THz, but reasonable results may be found at 1.75–2.1 THz and 2.4–2.75 THz.

**Figure 15:**
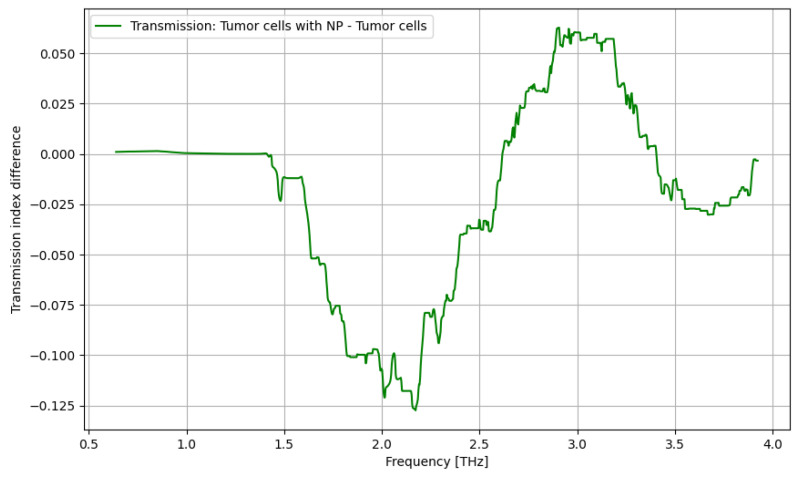
Transmission signal enhancement for tumor cells by use of nanoparticles.

In Figure 15, contrast enhancement by using nanoparticles is presented in terms of identifying tumor cells. The most relevant frequency domain is 2.6–3.25 THz.

**Figure 16:**
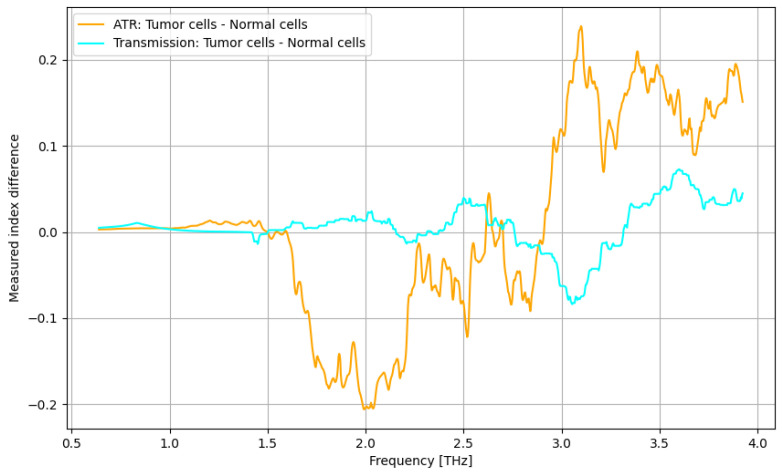
Comparative signal difference between the tumor cells and normal cells.

**Figure 17:**
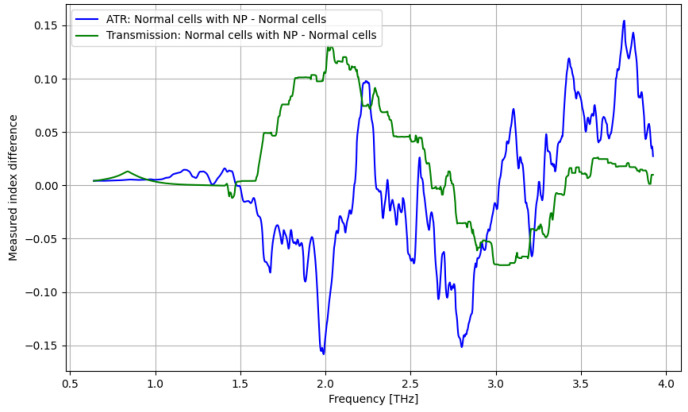
Comparative signal difference between the tumor cells and normal cells, nanoparticle addition.

**Figure 18:**
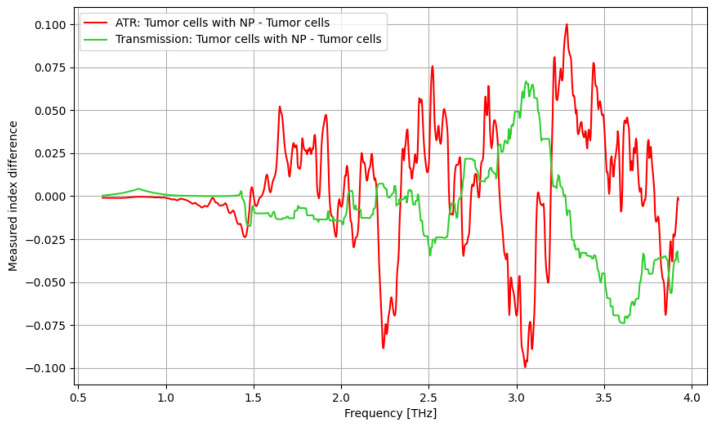
Comparative analysis of the influence of nanoparticles on cancer cell identification.

**Figure 19:**
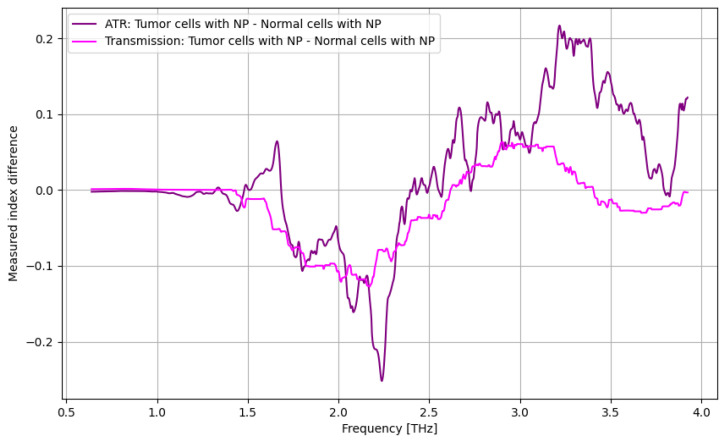
Comparative analysis of the influence of nanoparticles for cancer cell discrimination from normal cells.

The primary goal of the analysis was to determine the efficacy of nanoparticles in identifying normal and tumorous cells, as well as enhancing the distinction between them. Given the non-normal distribution of our data, we utilized non-parametric statistical methods for the entirety of the analysis. Moreover, the analysis was repeated in several structured steps for both the ATR and transmission data to ensure comparability. Accordingly, before describing the statistical procedure and related conclusions, some preliminary suggestive results occurring from the previously discussed spectroscopy analysis are presented in Figure 16, Figure 17, Figure 18 and Figure 19 as comparative differences between the signal intensity. In Figure 16, the signal difference between the tumor cells and normal cells is presented. It is obvious that the ATR analysis is more sensitive, mainly regarding 1.75–2.25 THz and 3–3.75 THz frequency domains. The transmission analysis becomes relevant only at higher frequencies for narrower domains. In Figure 17, the influence of nanoparticles on normal cell identification is presented. As regards the ATR analysis, some sensitive narrow frequency domains can be identified, e.g., 2, 2.25, 2.75, 3.2, 3.4, and 3.75 THz, but from a technical point of view, it is difficult to use in medical practice, such as tailored frequencies. As regards the transmission analysis, here, we can identify a reasonably larger domain of frequency, 1.75–2.5 THz, where the effect of nanoparticles is beneficial. 

In Figure 18, the influence of nanoparticles on cancer cell identification is presented. Regarding the ATR analysis, a behavior similar to that presented in Figure 17 can be identified with some sensitive narrow frequency domains, e.g., 1.75, 2.25, 2.5, 3, and 3.25 THz. As regards the transmission analysis, here, we can also identify a reasonably larger domain of frequency, 2.75–3.25 THz, where the effect of nanoparticles is beneficial. In fact, adding nanoparticles moves the sensitive domain towards larger frequencies, as shown in Figure 17 and Figure 18.

Finally, the most relevant analysis is related to comparing the spectroscopy process with and without nanoparticles when discriminating between cancer cells and normal cells (Figure 19). The ATR procedure is clearly superior to the transmission procedure for the frequency domain of 3.25–3.75 THz; in the rest, the difference is negligible. However, such interpretations of images are purely intuitive, which is why statistical analysis is compulsory, as presented below.

The first step in the statistical analysis was towards conducting multiple pairwise comparisons, including the support medium (without cells) versus the support medium with nanoparticles, normal cells versus normal cells with nanoparticles, and, respectively, tumor cells versus tumor cells with nanoparticles. Additional comparisons were made between normal and tumor cells, with and without nanoparticles. These initial tests helped determine if nanoparticles affected the spectroscopic measurements and if their impact varied between different types of cells. 

A critical part of our analysis was the “difference of differences” test, designed to directly assess whether the addition of nanoparticles more effectively enhanced the differentiation between tumor and normal cells compared to when nanoparticles were not used. This involved calculating the differences in measurements (tumor vs. normal) with and without nanoparticles and applying the statistical test to the difference in these differences, thus providing an evaluation of the impact of nanoparticles on enhancing cellular differentiation in spectroscopic data.

To further dissect the effects of nanoparticles at specific frequencies, we segmented our data into wavenumber intervals of length 1, performing the Wilcoxon test [86] within each interval for the same set of comparisons and for the difference in differences. This approach allowed us to pinpoint the specific frequency ranges where nanoparticles might exert significant effects, thereby identifying the spectral regions that benefit most from the addition of nanoparticles. 

In the final step of our analysis, we compared the effectiveness of ATR and transmission spectroscopy methods. This involved further tests comparing differences within each method, focusing on tumor versus normal cells, both with and without nanoparticles, and on the difference between cells of each type under the addition of nanoparticles. This comparison aimed to identify which spectroscopy method more effectively differentiated between the sample types under various conditions, enhancing our understanding of the optimal approaches in spectroscopic analysis.

The core of our statistical analysis relied on the Wilcoxon signed-rank test, a non-parametric method well suited for comparing paired samples, first introduced by Frank Wilcoxon [86]. This test was particularly appropriate for our study, where each set of measurements was inherently paired, as each pair of data points corresponded to the same wavelength but different experimental conditions (e.g., with and without nanoparticles). The Wilcoxon test does not assume a normal distribution of the data, making it ideal for our dataset, which exhibited non-normal characteristics.

The Wilcoxon signed-rank test evaluates differences between paired observations by considering both the magnitude and direction of these differences. First, the absolute differences are calculated to assess the magnitude. Each difference is then ranked, with larger differences receiving higher ranks. Crucially, these ranks are adjusted to reflect the direction of the differences: positive differences and negative differences are summed separately. The test statistic is derived from the smaller of these two sums, indicating whether positive or negative differences predominate. This statistic is compared against a reference distribution under the null hypothesis that the median difference between pairs is zero. In [87], examples of the use of this test for “Paired-Sample Hypotheses” in the context of biostatistics underscores its suitability for our analysis where changes in spectroscopic profiles due to different experimental conditions are examined. By providing *p*-values, the Wilcoxon test quantifies the probability that the observed differences between paired samples could occur under the null hypothesis. A *p*-value lower than 0.05 indicates a statistically significant result, suggesting that the observed differences are unlikely to have occurred by chance at the 95% confidence level. This threshold helps us determine whether the application of nanoparticles significantly alters the spectroscopic profiles of the samples. The results of these tests, indicating the *p*-values of differences observed in our experiments, are comprehensively detailed in the accompanying tables.

Hence, for both measurement types, i.e., ATR and transmittance, the signal difference between tumor cells and normal cells, with and without nanoparticles, was calculated and statistically validated for the entire frequency domain. Secondly, the statistical analysis was particularized on four frequency domains to identify the most appropriate frequency domain for medical use.

The statistical results for ATR measurements for the entire frequency domain are presented in Table 1. The homolog analysis for the four frequency domains is presented in Table 2.

As a general observation, if relating to the entire frequency domain, the ATR analysis can differentiate only the normal cells vs. normal cells with nanoparticles, an aspect clearly insufficient for a pertinent analysis; that is why the statistical analysis on narrower frequency domains is necessary. As regards the frequency domain relevance, we estimated the following:

- At low frequencies (0–1 THz), one can see a clear difference when adding nanoparticles in all situations and for all sample types.
- At 1–2 THz, the addition of nanoparticles has a significant effect only on normal cells.
- The domain of 2–3 THz frequencies is not suitable for the proposed analysis.
- At 3–4 THz, the difference between normal cells and tumor cells is significant only when adding nanoparticles, an aspect also suggested by Figure 19.

As regards transmission measurements, the statistical results for the entire frequency domain are presented in Table 3. The homolog analysis for the four frequency domains is presented in Table 4.

As a general observation, the transmission measurement techniques are more relevant than the ATR techniques when addressing the entire frequency domain. As regards the frequency domains’ relevance for transmission measurements, we estimated the following:

- At low frequencies (0–1 THz), we can see a difference when adding nanoparticles in all situations and for all sample types.
- At 1–2 THz, the addition of and for all sample types has a significant effect on tumor cells–opposite to the ATR results.
- At 2–3 THz, the addition of and for all sample types has a significant effect on normal cells, and the difference between normal cells and tumor cells is significant only when adding nanoparticles.
- At 3–4 THz, the difference between normal cells and tumor cells is significant only when adding nanoparticles.

Correlating the results at low frequencies (0–1 THz) for both ATR and transmission measurement techniques, it is obvious that, even if reduced in our case, the effect of adding nanoparticles is visible. This explains why many research studies presented in the literature refer to this peculiar frequency domain, which is suitable for using contrast agents derived from MRI procedures. 

Finally, the statistical analysis referred to the “difference of differences” for the entire frequency domain, where the following applied:

d_1_ = ATR/tumor cells—ATR/normal cells;

d_2_ = Transmittance/tumor cells—transmittance/normal cells; 

*p*-value = 0.695;

d_3_ = ATR/normal cells with nanoparticles—ATR/normal cells;

d_4_ = Transmittance/normal cells with nanoparticles—transmittance/normal cells

*p*-value = 0.152;

d_5_ = ATR/tumor cells with nanoparticles—ATR/tumor cells;

d_6_ = Transmittance/tumor cells with nanoparticles—transmittance/tumor cells

*p*-value = 0.110;

d_7_ = ATR/tumor cells with nanoparticles—ATR/normal cells with nanoparticles;

d_8_ = Transmittance/tumor cells with nanoparticles—transmittance/normal cells with nanoparticles. 

*p*-value = 0.012 *.

The most relevant result is the *p*-value = 0.012 when generally comparing ATR and transmission measurement procedures for a broader frequency domain. The differentiation between normal and tumor cells in the presence of contrast agents is superior when using the ATR procedure, an aspect noticed in the last few years by other research results presented in the literature, as in [80]. 

The results for the four frequency domains are presented in Table 5.

We noticed the following:

- At low frequencies (0–1 THz), the ATR measurement procedure is generally superior to the transmission measurement procedure in all cases when nanoparticles are added, but the difference between them is low.
- At 1–2 THz, the ATR measurement procedure is superior to the transmission measurement procedure in most cases, except for validating tumor cells with nanoparticles, where they practically present the same results. This result is consistent with the observations from Figure 18 and Figure 19.
- At 2–3 THz, the ATR measurement procedure is superior to the transmission measurement procedure only in the case of discriminating tumor cells from normal cells by using nanoparticles; in rest, they practically present the same results. The result is consistent with the observations from Figure 19.
- At 3–4 THz, the ATR measurement procedure is superior to the transmission measurement procedure only in the case of discriminating tumor cells in general by using nanoparticles; in the rest, they practically present the same results. The result is consistent with the observations from Figure 18 and Figure 19.

Accordingly, our methodical approach effectively evaluated how nanoparticles enhance the spectroscopic differentiation of cell types via ATR and respectively transmission methods. This rigorous analysis, supported by the Wilcoxon signed-rank test across various conditions, may be expanded for other types of contrast nanoparticles and may lay a strong foundation for advancing THz spectroscopy techniques for oncological diagnosis.

The development of contrast agents dedicated to THz spectroscopy remains a difficult task and the simplest approach is to test and eventually chemically–physically adapt contrast agents already medically certified and in use, e.g., the ones for MRI. The contrast agent that we described in the paper is in line with this purpose. Another example of a typical contrast agent is derived from Fe_3_O_4_, but some recent research studies have emphasized the toxic effects of Fe_2_O_3_ nanoparticles on the liver and lung tissue [88], aspects that confirms the challenge in finding sustainable contrast agents. As long as all imagistic techniques are working in narrow bands, we expect THz spectroscopy to be also used in certain narrow bands and, respectively, the effect of a certain contrast agent to be identified in a specific narrow band, exactly the case described in the paper. It is obvious that, due to their chemical–physical parameters, which limit their absorption and/or transmission properties, the nanoparticles’ behavior is clearly influenced by the frequency domain—which must be a priori chosen to be larger for a pertinent analysis—and in our case, till 4 THz, and the optimal response of nanoparticles can be found only in certain narrow bands, as we statistically demonstrate in the paper.

It is a certitude that THz spectroscopy and imagistic methods will be further developed for medical use, even if, for the moment, almost all studies refer to living cells, or tissues as surgical resected specimens, including for gastric cancer [89,90,91], and not living subjects, even if a direct application is tested for skin cancer [92,93], which is still not yet clinically homologated. The biggest impediment in developing a THz spectroscopy and imagistic method for gastric or colorectal cancer, to be directly used for clinical medicine on patients, is related to the successful development of a minimally invasive and flexible endoscopic system associated with THz spectrometry, but the actual technological limitations in terms of miniaturizing the THz sensors justifies this legitimate desire to wait for further technical advances [94,95,96]. Accordingly, we are sure that an increase in the THz frequency domain and the development of new contrast agents will allow a balance with the technological development of miniaturized THz sensors for THz endoscopy in clinical oncology.

## 4. Conclusions

The paper describes the statistical analysis of the response of cancer cells (a human gastric adenocarcinoma cell line) and normal human gastric cell lines to broadband terahertz radiation up to 4 THz, a frequency range considered optimal for biomedical applications. The investigation was conducted both with and without the use of nanostructured contrast agents related to MRI contrast agents based on gadolinium oxide and carboxymethylcellulose. The THz spectroscopy analysis was comparatively performed under the ATR procedure and transmission measurement procedure.

As regards the ATR procedure, the highest discrimination between tumor cells and normal cells by using nanoparticles may be noticed mainly in the frequency domain of 3.25–3.5 THz, even if the differentiation can be extended from 2.25 to 3.75 THz.

As regards the transmission measurement procedure, the highest discrimination between tumor cells and normal cells by using nanoparticles may be noticed mainly in the frequency domain of 2.6–3.25 THz.

When comparing the spectroscopy process in terms of signal enhancement by the addition of nanoparticles in order to discriminate between cancer cells and normal cells, the ATR procedure is clearly superior to the transmission procedure for the frequency domain of 3.25–3.75 THz; in the rest, the difference is negligible.

The statistical analysis was conducted towards multiple pairwise comparisons, including the support medium (without cells) versus the support medium with nanoparticles, normal cells versus normal cells with nanoparticles, and, respectively, tumor cells versus tumor cells with nanoparticles. Additional comparisons were made between normal and tumor cells, with and without nanoparticles. Another stage of the analysis was dedicated to the “difference of differences” test, designed to directly assess whether the addition of nanoparticles more effectively enhanced the differentiation between tumor and normal cells compared to when nanoparticles were not used. *p*-values were calculated to quantify the probability that the observed differences between paired samples could occur under the null hypothesis, where changes in spectroscopic profiles due to different experimental conditions were emphasized and were validated by the Wilcoxon signed-rank test.

We notice that at low frequencies (0–1 THz), the ATR measurement procedure is in general superior to the transmission measurement procedure in all cases when nanoparticles were added, but the difference between them is low; at 1–2 THz, the ATR measurement procedure is superior to the transmission measurement procedure in most cases, except for the validation of tumor cells with nanoparticles; at 2–3 THz, the ATR measurement procedure is superior to the transmission measurement procedure only in the case of discriminating tumor cells from normal cells by using nanoparticles, and in the rest, they practically present the same results; and at 3–4 THz, the ATR measurement procedure is superior to the transmission measurement procedure only in the case of discriminating tumor cells in general by using nanoparticles, and in the rest, they practically present the same results.

When generally comparing the ATR procedure and transmission measurement procedure for a broader frequency domain, the differentiation between normal and tumor cells in the presence of contrast agents is superior when using the ATR procedure, an aspect noticed in the last few years by other research results presented in the literature. On the other hand, the THz contrast enhancement by using contrast agents derived from MRI-related contrast agents, as presented in our case, leads only to limited benefits only for narrow THz frequency domains, an aspect that must encourage future research in developing other concepts of contrast agents for THz medical imaging. 

The biggest impediment in developing a THz spectroscopy and imagistic method for gastric or colorectal cancer is related to the successful development of a minimally invasive and flexible endoscopic system associated with THz spectrometry, but we are sure that an increase in the THz frequency domain and the development of new contrast agents will allow a balance with the technological development of miniaturized THz sensors for THz endoscopy in clinical oncology.

## Figures and Tables

**Table 1:** Statistical analysis of ATR measurements for the entire frequency domain.

Sample Type	*p*-Value
Support medium (without cells) vs. support medium with nanoparticles	0.000 *
Normal cells vs. normal cells with nanoparticles	0.027 *
Tumor cells vs. tumor cells with nanoparticles	0.086
Normal cells vs. tumor cells	0.227
Normal cells with nanoparticles vs. tumor cells with nanoparticles	0.731
Difference in differences	0.487

* very relevant.

**Table 2:** *p*-values related to ATR measurements vs. frequency domains.

Sample Type	0.06–1 THz	1–2 THz	2–3 THz	3–4 THz
Normal cells vs. normal cells with nanoparticles	0.000 *	0.0012 *	0.145	0.016 *
Tumor cells vs. tumor cells with nanoparticles	0.000 *	0.517	0.106	0.0000 *
Normal cells vs. tumor cells	0.000 *	0.021 *	0.609	0.629
Normal cells with nanoparticles vs. tumor cells with nanoparticles	0.000 *	0.394	0.497	0.043 *
Difference in differences	0.000 *	0.168	0.981	0.243

* very relevant.

**Table 3:** Statistical analysis of transmission measurements for the entire frequency domain.

Sample Type	*p*-Value
Support medium (without cells) vs. support medium with nanoparticles	0.342
Normal cells vs. normal cells with nanoparticles	0.0000 *
Tumor cells vs. tumor cells with nanoparticles	0.0021 *
Normal cells vs. tumor cells	0.0000 *
Normal cells with nanoparticles vs. tumor cells with nanoparticles	0.123
Difference in differences	0.0000 *

* very relevant.

**Table 4:** *p*-values related to transmission measurements vs. frequency domains.

Sample Type	0.06–1 THz	1–2 THz	2–3 THz	3–4 THz
Normal cells vs. normal cells with nanoparticles	0.0000 *	0.971	0.0033 *	0.0000 *
Tumor cells vs. tumor cells with nanoparticles	0.0000 *	0.0062 *	0.666	0.0107 *
Normal cells vs. tumor cells	0.0000 *	0.0044 *	0.065	0.061
Normal cells with nanoparticles vs. tumor cells with nanoparticles	0.0034 *	0.0040 *	0.0000 *	0.0000 *
Difference in differences	0.0000 *	0.051 * (limit)	0.055 * (limit)	0.0000 *

* very relevant.

**Table 5:** “Difference of differences” vs. frequency domains.

Frequency	0.06–1 THz	1–2 THz	2–3 THz	3–4 THz
d_1_–d_2_	0.097	0.0012 *	0.159	0.6099
d_3–_d_4_	0.0039 *	0.0003 *	0.544	0.7877
d_5_–d_6_	0.0033 *	0.7009	0.7134	0.0015 *
d_7_–d_8_	0.0000 *	0.0000 *	0.0000 *	0.0000 *

* very relevant.

## Data Availability

Data are contained within the article.
